# Use of cotinine biomarker in workers to detect green tobacco sickness^*^

**DOI:** 10.1590/1518-8345.3141.3194

**Published:** 2019-10-14

**Authors:** Marta Regina Cezar-Vaz, Marcia Casaril dos Santos Cargnin

**Affiliations:** 1 Universidade Federal do Rio Grande, Rio Grande, RS, Brasil.; 2 Universidade Regional Integrada do Alto Uruguai e das Missões – Campus Frederico Westphalen, Frederico Westphalen, RS, Brasil.

**Keywords:** Biomarkers, Cotinine, Occupational Diseases, Tobacco, Nicotine, Rural Workers, Biomarcadores, Cotinina, Doenças Ocupacionais, Tabaco, Nicotina, Trabalhadores Rurais, Biomarcadores, Cotinina, Enfermedades Ocupacionales, Tabaco, Nicotina, Trabajadores Rurales

## Abstract

**Objective:**

using the urinary cotinine biomarker to verify the occurrence of green tobacco sickness in workers who cultivate Burley tobacco.

**Method:**

paired case-control study, based on smoking status and on the 1:4 ratio, with participation of 20 case workers and 91 controls. Data collection included household surveys and urine collection for cotinine examination. Student’s T-Test, the Mann-Whitney test, Pearson’s chi-square or Fisher’s exact tests were used.

**Results:**

of the 23 suspected cases, 20 showed elevated levels of cotinine, signs and symptoms of headache, skin irritation, nausea, sickness and general malaise, especially in the morning. Most had worked with tobacco that was wet from the morning dew and when the weather was warm.

**Conclusion:**

there are signs suggestive of green tobacco sickness in Burley tobacco workers. The action of health professionals is necessary for the development of health promotion and preventive actions addressing work-related illness.

## Abstract (pt)

**Objetivo:**

verificar, por meio da tecnologia de biomarcador de cotinina urinária, a ocorrência da doença da folha verde do tabaco em trabalhadores que cultivam tabaco do tipo Burley.

**Método:**

estudo caso-controle pareado, com base no *status* tabágico e na proporção 1:4, com participação de 20 trabalhadores-caso e 91 controles. Para a coleta de dados foi realizada entrevista por meio de inquérito domiciliar e coleta de urina para exame de cotinina. Foram utilizados os testes *t* de Student, Mann-Whitney, qui-quadrado de Pearson ou exato de Fisher.

**Resultados:**

dos 23 casos suspeitos, 20 apresentaram níveis elevados de cotinina, sinais e sintomas de dor de cabeça, irritação de pele, náusea, enjoo e mal-estar geral, principalmente pela manhã. A maioria trabalhou com tabaco molhado decorrente do sereno e o clima estava quente.

**Conclusão:**

verificam-se sinais sugestivos da doença da folha verde do tabaco nos trabalhadores do tabaco Burley. Faz-se necessária a atuação de profissionais de saúde no desenvolvimento de ações promotoras de saúde e preventivas sobre essa doença relacionada ao trabalho.

## Abstract (es)

**Objetivo:**

certificar, por medio de la tecnología de biomarcador de cotinina urinaria, la ocurrencia de la Enfermedad del Tabaco Verde en trabajadores que cultivan tabaco del tipo Burley.

**Método:**

estudio caso-control pareado, con base en el *status* tabáquico y en la proporción 1:4, con la participación de 20 trabajadores-caso y 91 controles. Para la recopilación de datos se realizó una encuesta por medio de cuestionario domiciliar y de recolección de orina para análisis de cotinina. Se utilizaron las siguientes pruebas *t* de Student, Mann-Whitney, chi-cuadrado de Pearson o exacta de Fisher.

**Resultados:**

de los 23 casos sospechosos, 20 presentaron niveles elevados de cotinina, señales y síntomas de dolor de cabeza, irritación de la piel, náusea, mareo y malestar general, principalmente por la mañana. La mayoría trabajó con tabaco mojado por el sereno y en un clima caluroso.

**Conclusión:**

se certifican señales sugestivas de Enfermedad del Tabaco Verde en los trabajadores del tabaco Burley. Se hace necesaria la actuación de profesionales de la salud en el desarrollo de acciones promotoras de salud y preventivas de esta enfermedad relacionado con el trabajo.

## Introduction

Studies have revealed the close relationship between tobacco production modes and health problems and demonstrated the conditions that negatively influence the health status of tobacco-producing families^1-3^. The activities developed in tobacco production expose workers to the risk of illness, such as osteoarticular disorders, diseases caused by solar radiation, acute and chronic intoxications caused by pesticides, respiratory disorders, mental diseases and green tobacco sickness (GTS)^3-4^

The health risks associated with tobacco production were registered in 1713 by Bernadino Ramazzini, with the description of signs and symptoms such as headache and gastrointestinal disorders in Italian tobacco producers^5^. Only in 1970, in Florida – United States of America (USA), GTS was reported as a specific disease of rural tobacco workers^6^

GTS is an acute intoxication triggered by dermal absorption and nicotine inhalation, with the following signs and symptoms: nausea, vomiting, weakness, dizziness, headache, insomnia and loss of appetite^3,7-8^ These signs and symptoms affect workers mainly during planting, cultivation, harvesting, curing and baling^9-10^, occurring especially when their clothes or the tobacco leaves become wet with rain, dew or sweat^5^.

The morbidity of GTS affects almost a quarter of tobacco workers^11^. Workers are diagnosed with it based on their history of exposure to tobacco culture, presence of signs and symptoms of acute intoxication and abnormal cotinine dosage^8,12-13^.

The absorbed nicotine is bio-transformed into cotinine, its main metabolite and can be detected in the various biological fluids of individuals exposed to tobacco, such as urine, saliva and blood, having a biological half-life of about 20 hours^5,8^. In this sense, one of these biomarkers should be used to verify the presence of GTS^14^.

In Brazil, the presence of GTS was confirmed for the first time in the Agreste Alagoano region, in 2007^10^, by testing the workers’ urinary cotinine using the High Performance Liquid Chromatography method (HPLC). This method is the one adopted by most national and international studies, seeing as it is specific and its detection limits are lower^15^. Similarly, in 2008, GTS was confirmed in workers from a municipality in the Vale do Rio Pardo region, in Rio Grande do Sul (RS), in a case-control study^8^.

The need for this study was based on the evidence found in a research project^16^ which identified, using self-reports, the presence of signs and symptoms during the tobacco production process, especially in the stages of harvesting and preparation of the leaves, which would be suggestive of GTS. In addition, there is a need to deepen the knowledge on this theme in workers who cultivate Burley tobacco, since there is a limited number of studies on this theme in Brazil.

This need is also associated with the evidence found in a study^11^ in which it was identified that the mature leaves of non-Virginia tobacco (Burley among them) contain about three to four times more nicotine compared to Virginia tobacco leaves, with which the majority of existing studies have been developed. Thus, the present research study, with Burley tobacco workers, should provide comparisons between the other types of tobacco produced in Brazil, since some stages of the work process also differ. Tobacco of the Burley, Common and Virginia varieties is cultivated in the Southern region of Brazil. The cultivation processes of the different varieties are the same, with the exception of the stage of harvesting of the leaves, curing and final preparation.

In this sense, studies on GTS in workers and families involved in the tobacco production process become relevant for the development of interdisciplinary and intersectoral actions to promote the health of the rural population, from an environmental perspective. Health and disease are environmental phenomena in the relationships with nature and in the interrelationship between all beings^17^. These (inter)relationships develop into relationships of adequacy or inadequacy to the environment, which may cause imbalance, in the case of the present study, in human beings when they become ill, and also in the land, with the planting of tobacco.

Thus, as environmental health is a field of nursing practice, it is necessary that professional nurses acquire sufficient knowledge about the health-work-environment process, in order to develop actions directed to these workers in an individual and collective manner. In addition, there is a need for these professionals to become each time more inserted in the different spaces related to workers’ health, especially to that of rural workers, so as to obtain scientific subsidies for the improvement of working conditions, which would minimize health problems.

In this sense, the objective of this study is to use the urinary cotinine biomarker to verify the occurrence of green tobacco sickness in workers who cultivate Burley tobacco.

## Method

This is a case-control study, represented by the 1:4 ratio during the Burley tobacco harvesting period, in the months of December 2016 to January 2017 (2016/2017 crop) and, to complement the sample, December 2017 (crop 2017/2018), with rural workers who cultivated Burley tobacco, in a municipality located in the northwest region of the state of Rio Grande do Sul, Brazil. Four samples were collected in December 2017, regardless of the environmental conditions; even if they were not the same as in the previous harvest, the work process was the same, and the environmental variability was minimal, having not significantly influenced the final results.

The following inclusion criteria were adopted: rural workers who were cultivating Burley tobacco; who were in the harvesting stage during the data collection period; who had worked with Burley tobacco farming in the seven days preceding the interview; who had not been exposed to other varieties of tobacco in the seven days preceding the interview; who had not been exposed to pesticides in the seven days preceding the interview; who were 18 years of age or older, and who were willing to provide a urine sample. The exclusion criteria were workers who were pregnant and those with self-reported mental/psychological disorders that made it impossible for them to understand the questions of the research instrument.

A total of 159 people involved with tobacco cultivation in the 2016/2017 and 2017/2018 crop were identified with the aid of community health agents, with 100% coverage of the Family Health Strategy and confirmation by the technicians of Emater/RS-Ascar,a public institution associated with the State Secretariat of Agriculture, Livestock and Supply of each state that provides technical assistance and rural extension to rural workers. The sample size calculation was performed on the WinPEPI program *(*Programs for epidemiologists for Windows), version 11.43, and based on national^8,10^ and international^14^studies. Considering a 5% significance level, prevalence of the estimated GTS at 20%, ratio of four controls for each case and 4.5 minimum odds ratio, the minimum number would be 19 cases and 76 controls, totaling 95 workers. Of the 159 tobacco workers, 37 did not participate, because they did not meet the inclusion criteria.

According to studies^8,10^ conducted in Brazil, workers who reported signs and symptoms of acute intoxication (headache, nausea, vomiting, dizziness or weakness) 48 hours prior to the collection of a urine sample were considered suspected cases. The suspected cases with cotinine level above the reference values established by the laboratory were confirmed, these being: <20 ng/mL (nanogram per milliliter) for non-smokers; from 20 to 50 ng/mL for passive or occasional smokers; >50 ng/mL for smokers, detected by the urine test. The controls were workers who did not show any signs and symptoms of acute intoxication (headache, nausea, vomiting, dizziness or weakness) in the seven days preceding the interview.

Cases and controls were paired based on smoking status, because tobacco consumption can reduce the occurrence of GTS^12^due to the constriction of the vessels caused by consumption, metabolic adaptation or the tolerance acquired under the long-term effects of nicotine^18^. The individuals who smoked any type of tobacco every day for at least six months were considered smokers^19^.

Although the questionnaire that subsided the preparation of the instrument has already been applied to another population of workers, a pilot test of the collection instrument was performed with a group of ten workers – selected by convenience –, to ensure its validity, accuracy and reproducibility, as well as to identify any possible flaws in its wording and measure the duration of its application. The selection by convenience and these instruments were not part of the final sample. With the application of the test, it was possible to improve the writing of some questions, making them more understandable and objective for the workers; in addition, one question was excluded, and three others were included.

Household surveys were conducted for the collection of data, using an instrument adapted from other research^8^, that includes identification data, sociodemographic variables, GTS, exposure to tobacco, individual characteristics, smoking status, characteristics of alcohol consumption, exposure to pesticides and other exposures contained in 75 questions. Data and urine collection were performed by the researchers and seven research assistants, previously selected and trained, who went to the worker’s at the time scheduled by telephone in most cases, carrying a urine collection kit containing a thermal box, gel pack, disposable gloves, urine collector, numerical identification tags to put on the vials and control forms for each worker. After the interview, a polyethylene vial was provided for the collection of the urine samples, along with instructions on the collection, handling and storage of the sample in a refrigerator. It was also clarified that the next day, the researchers would come back for the urine sample, which would have to be the first in the morning.

Urine samples were collected to determine the cotinine levels of case workers and controls, kept frozen in an ultra-low freezer (-70 ºC) and sent to the reference toxicology and pharmacology laboratory for analysis. The verification of the dosage of cotinine in urine samples is one of the parameters for the verification of GTS, and the HPLC method with ultraviolet detector was used for this purpose.

The method used by the same laboratory of this research project is described in a study^10^, while the validation of the method for identification of cotinine in urine using HPLC is also reported in another study^15^, where the instrumentation and chromatographic conditions of all chemical products followed the HPLC grade standard, containing high-efficiency liquid chromatography, equipped with an isocratic pump, ultraviolet detector, de-aerification and manual injection system. The chromatographic separations were performed using a reverse phase column. The column was protected by a guard column and kept at 22±2ºC. The mobile phase was a mixture of ultrapure water: methanol: 0.1 M (Molarity) sodium acetate: acetonitrile, containing 1 mL of 0.034 M citric acid and 5.0 mL of triethylamine, added for each liter of solution. The method’s detection limit was 5 ng/mL, and the quantification’s limit was 10 ng/mL. To perform the analyses, the sample was prepared with 2.0 mL of urine treated with 25 μL (microliters) of 10 M sodium hydroxide and 100 μL of internal standard, and 4.0 mL of dichloromethane. In the organic phase, they were dried with nitrogen and by being kept at room temperature. Subsequently, 100 μl of the mobile phase were added and 20 μl were injected in HPLC^10,15^.

It is worth noting that the use of biomarkers is an increasingly frequent tendency to help prove the diagnosis and assist in the prognosis of the diseases. Thus, the combination of clinical presentation with cotinine level measurement allows more accurate estimates, excluding other confounding clinical hypotheses, mainly intoxications related to the work process.

The study data were analyzed on the Statistical Package for Social Sciences (SPSS), version 21.0. The quantitative variables were described by mean and standard deviation or median and interquartile range. The qualitative variables were described by absolute and relative frequencies. To compare means, Student’s T-Test was applied. In case of asymmetry, the Mann-Whitney test was used. To compare proportions, Pearson’s Chi-square test or Fisher’s exact test were used. The significance level adopted was 5% (p<0.05). The internal consistency of the instrument was analyzed with Cronbach’s Alpha, and the value obtained was 0.723.

This study was approved by the Research Ethics Committee of Universidade Regional Integrada do Alto Uruguai e das Missões (URI), under Opinion No. 1.791.798. The research project was approved by the Ethics Committee for Research in the Health Field (CEPAS) of the Federal University of Rio Grande (FURG) under Opinion No.1.887.270, in January 2017.

## Results

Twenty-three Burley tobacco workers were defined as suspected cases, and of these, 20 were confirmed by the result of the urinary cotinine test. Of the confirmed cases, 11 were men; their mean age was 43.5 years old and they were mostly white (18; 90%), with 7.3 years of education on average. As for the ingestion of alcoholic beverages, 14 (70.0%) reported using them, while five (35.7%) reported their consumption frequency to be from one to two times a week, as described in Table 1.

**Table 1:** – Characteristics of case workers and controls who cultivate Burley tobacco. Taquaruçu do Sul, RS, Brazil, 2016-2017

Variables	Cases (n=20)	Controls (n=91)	p*
	n (%)	n (%)	
Sex			0,337
Male	11 (55,0)	63 (69,2)	
Female	9 (45,0)	28 (30,8)	
Age	43,5±12,9	47,3±12,3	0,214
Ethnicity			1,000
White	18 (90,0)	78 (85,7)	
Mixed	2 (10,0)	13 (14,3)	
Education level (years)	7,3±2,7	7,06±3,33	0,713
Alcohol consumption	14 (70,0)	72 (79,1)	0,385
Frequency of use			0,584
Daily	3 (21,4)	10 (13,9)	
Less than 1x per week	4 (28,6)	20 (27,8)	
1-2x per week	5 (35,7)	34 (47,2)	
3-4x per week	2 (14,3)	4 (5,6)	
5-6x per week	0 (0,0)	4 (5,6)	

*Comparison of means by Student’s T test and of proportions by the chi-square test or Fisher’s exact test.

Table 1 shows that the disease was not associated with sex, age, ethnicity, education level, use and frequency of alcoholic beverages.

In relation to the association of the workers with the property where they reside, 18 (90.0%) reported to own it and two (10.0%) were tenants. Seven develop, as main activities, the production of milk and tobacco (35.0%), respectively; three (15.0%) were homemakers (women); two (10.0%) reported swine farming; and one (5%) reported maize cultivation.

The workers began to show signs and symptoms of intoxication, mostly in the morning (11; 55.0%); six (30.0%) in the evening; and three (15.0%) in the afternoon. Of these, only one (5.0%) sought the hospital, having stayed under observation for approximately 50 minutes to receive intravenous medication.

The signs and symptoms reported were: headache in ten occurrences (50.0%); skin irritation in eight (40.0%); nausea and nausea in seven (35.0%); malaise in six (30.0%); excessive sweating in four (20.0%); weakness in four (20.0%); dizziness in three (15.0%); abdominal pain in three (15.0%); eye irritation in three (15.0%); vomiting in two (10.0%); increased salivation in one (5.0%); very dry mouth in one (5.0%); blurred vision in one (5.0%); bitter taste in mouth/throat burning in one (5.0%) and diarrhea in one occurrence (5.0%). These signs and symptoms lasted a median of 300 (135-2520) minutes – minimum of 15 minutes and maximum of seven days (the latter refers to skin irritation). Seven participants (35.0%) were still feeling the signs and symptoms on the day of the interview.

Regarding the day they started feeling the signs and symptoms, 14 (70.0%) reported that the weather was hot and they had been working with wet tobacco; nine (64.3%) reported the presence of morning dew; and seven (50.0%) of rainfall, as shown in Table 2.

**Table 2:** – Socioenvironmental conditions of individuals (cases and controls) who cultivate Burley tobacco. Taquaruçu do Sul, RS, Brazil, 2016-2017

Variables	Cases (n=20)	Controls (n=91)	p*
	n (%)	n (%)	
Presence of wound/cut on hands	1 (5,0)	^†^	
Weather^‡^			
Warm	14 (70,0)	^†^	
Sunny	13 (65,0)	^†^	
Humid	6 (30,0)	^†^	
Rainy	5 (25,0)	^†^	
Cloudy	2 (10,0)	^†^	
Worked with wet tobacco^‡^	14 (70,0)	63 (69,2)	1,000
Morning dew	9 (64,3)	45 (71,4)	0,748
Rain	7 (50,0)	30 (47,6)	1,000
Clothes got wet^‡^	17 (85,0)	74 (81,3)	1,000
Sweat	13 (76,5)	52 (70,3)	0,769
Morning dew	10 (58,8)	30 (40,5)	0,272
Rain	2 (11,8)	15 (20,3)	0,513
Changed clothes	8 (47,1)	42 (56,8)	0,650

*Chi-Square or Fisher’s exact tests; ^†^The workers who did not show signs and symptoms of the disease were not questioned about it; ^‡^Multiple answers

While working with tobacco, 17 (85.0%) workers reported having gotten their clothes wet, and only eight (47.1%) changed them. These and other data are described in Table 2. The data collection period, which ranged from December 7, 2016 to January 19, 2017, was a period of frequent rainfall, totaling approximately 261 mm (millimeters) of rainfall; in the collection carried out in December 2017, on the other hand, there was no rainfall, but temperatures remained high.

The tobacco workers reported using the following pieces of clothing to work: pants, 17 (85.0%); hat, 16 (80.0%); long-sleeved shirt, 14 (70.0%); boots, 12 (60.0%) and socks, nine (45.0%), among others, as shown in Table 3. It is worth noting that one of the few Personal Protective Equipment (PPE) used, besides boots, were polyurethane gloves, reported by seven of them (35.0%), having been made available/distributed by the tobacco-producing company. There was a significant association between the disease and the use of socks.

**Table 3:** – Clothes/clothing and/or personal protective equipment used by workers (cases and controls) who cultivate Burley tobacco. Taquaruçu do Sul, RS, Brazil, 2016-2017

Variables	Cases (n=20)	Controls (n=91)	p*
	n (%)	n (%)	
Clothes/PPE^†^			
Pants	17 (85,0)	67 (73,6)	0,392
Hat	16 (80,0)	75 (82,4)	0,756
Long-sleeved shirt	14 (70,0)	47 (51,6)	0,213
Boots	12 (60,0)	55 (60,4)	1,000
Socks	9 (45,0)	14 (15,4)	0,006
Cap	7 (35,0)	22 (24,2)	0,474
Shoes/Sneakers/Ankle boots	7 (35,0)	23 (25,3)	0,543
Short-sleeved shirt	6 (30,0)	44 (48,4)	0,213
Slippers	4 (20,0)	28 (30,8)	0,490
Shorts	3 (15,0)	25 (27,5)	0,380
Sunscreen	2 (10,0)	7 (7,7)	0,664
Types of gloves			
Polyurethane	7 (35,0)	25 (27,5)	0,689
Cotton	1 (5,0)	1 (1,1)	0,329
Latex	1 (5,0)	3 (3,3)	0,554
Latex with cotton	2 (10,0)	5 (5,5)	0,607

*Chi-square or Fisher’s exact tests; ^†^Multiple answers

One of the inclusion criteria in this study was not having been exposed to pesticides in the last seven days. When questioned about the last time they had come into contact with pesticides, the median obtained was 30 (9-40) days.

The workers reported the time elapsed since their last contact with tobacco until the time they collected the first morning urine. The median obtained was 11.5 (10-15) hours for the case workers and 12 (10-15) hours for the control workers.

During the Burley tobacco harvesting period, 23 suspected cases were identified. Of these, three had undetectable cotinine levels, while 20 had abnormal levels. Table 4 shows the urinary cotinine dosage of the control workers, with a median of 98.5 (30-206.7) ng/mL. It should be noted that 61 non-smoking control workers and two passive smoking workers did not show the signs and symptoms of GTS, but their cotinine levels were above the reference values.

**Table 4:** – Median levels of urinary cotinine in workers (cases and controls) who cultivate Burley tobacco. Taquaruçu do Sul, RS, Brazil, 2016-2017

Variables	n	Median	P25-P75*	p^†^
Cases	20	114,9	(84,3-272,2)	0,092
Controls	91	98,5	(30-206,7)	
Smoker				0,222
Cases	1	1484	-	
Controls	8	184,9	(97,6-407)	
Non-smoker/former smoker				0,088
Cases	19	111,7	(83,5-248,9)	
Controls	83	89,2	(24-191)	

*P25-P75 = 25-75 percentiles; ^†^Mann-Whitney Test

In the urinary cotinine analysis, the smoking status was stratified (smoker versus non-smoker). There was no statistically significant difference between the mean levels of cotinine of the smoking (p=0.222) and non-smoking (p=0.088) case and control workers, as shown in Table 4.

## Discussion

This article aimed to verify the occurrence of GTS in workers who cultivate Burley tobacco, which contributes to approximately 14% of the total produced in the southern region of Brazil^20^. Burley tobacco has a dark hue and contains, on average, three to four times more nicotine than the Virginia variety^11^.

The production of tobacco developed in small farms involves the intensive use of the workforce of producing families at all stages of production, and some, such as the planting of seedlings and harvesting of the tobacco leaves, require the involvement of practically the entire family^21^. In this way, the participation of both men and women in the present study is revealed, mostly of men, in the group of cases as well as in the control group. These findings are similar to those of other studies developed in Brazil with the same methodology^8,10^. It is important to pay attention to women’s workload because, in addition to participating in the tobacco production process, they have to do housework as well as help their companions in other activities around the property, such as milking cows.

In a cross-sectional study^9^ carried out with tobacco workers, a higher prevalence of GTS was found in women (11.9%) compared to men (6.6%), which was related to biological differences, since women have greater dermal area (body volume) for the absorption of nicotine.

As for the workers’ age, both cases and controls are adults considered to be “mature”, contrary to the published national investigations, where the median age of the cases was 21 years old^10^ and their mean age was 33 years old^8^. In a cross-sectional study, age remained associated with GTS among men, and those who were between 30 and 39 years old were shown to be at higher risk^9^, which was related to the greater work intensity of young workers.

The signs and symptoms reported by the workers in this research project (headache, skin irritation, nausea, among others) were also described in other studies^1,8,10,14^. Similar symptoms were described in a study conducted in the region of Vale do Rio Pardo, RS, during the tobacco classification period, in which the main symptoms were nausea, headache, dizziness, abdominal discomfort and weakness^22^. Vomiting, nausea, dizziness, and headache are caused by the stimulation or inhibition of cholinergic receptors in the central nervous system, leading to the clinical manifestations^4^ described by the workers.

In this study, these manifestations lasted a median of five hours (from 15 minutes to seven days), but the mean duration described in the studies was from one to three days^12,14^, varying from 21 hours^8^ to 23 days^23^. Symptoms can start showing from a few minutes, since the contact with the worker’s skin, to hours later (3 to 17 hour interval)^5^.

The signs and symptoms of GTS are often confused with pesticide poisoning and heat exhaustion^6,8^. In this study, it can be stated that acute intoxication is not related to exposure to pesticides, because the harvesting stage implies in lower application of pesticides^8^, in addition to the workers being in contact with the plant for more than seven days at the time of sampling.

On the day the workers in this research project showed signs and symptoms of intoxication, they had worked with tobacco that was wet from the morning dew and rain, and the weather was hot. Soluble in water, the nicotine present in tobacco leaves is absorbed through the skin (hands, forearms, thighs, back and feet), and the water from the rain, dew or the worker’s perspiration present in the plant enhances its transdermal absorption^6,9-10^. This evidence was confirmed in a research project^10^ where an increase in the number of people with signs and symptoms of GTS in rainy days was identified.

In another study^24^ carried out in Malaysia, it was found that tobacco workers working under wet conditions had pallor, rash, and muscle weakness more frequently. The climate is an important factor for the development of GTS, since rainy and humid days especially increase the dermal absorption of nicotine, resulting in increased morbidity rates^23^.

The dew found on tobacco leaves usually soaks the workers’ clothes shortly after they start working^6^. In addition to the dew, the workers in this research project reported that their clothes got wet from the rain and sweat, but most do not change them – this is a condition associated with GTS, as wet clothes may increase the workers’ exposure to nicotine through dermal absorption^14^. For this reason, the use of PPE is indicated to reduce GTS^24^. In a case-control study conducted in eastern North Carolina, USA, it was shown that the use of rainwear while working with wet tobacco can significantly reduce the risk of GTS among workers^18^, but most of the time, protective equipment is not used, since it complicates the harvesting process^4^.

In this research project, it was possible to show that the workers wear hats, pants and long-sleeved shirts to work with tobacco. These provide sun protection only since they are not waterproof. The polyurethane gloves used by some workers provide protection against moisture only on the palms (palmar face and fingertips) and on the back of the hands, with nylon (polyamide) threads, favoring ventilation for greater thermal comfort, but are however not waterproof. Cotton gloves provide the least protection (78.5%), but are more comfortable and of low durability, while rubber gloves offer 93% protection^25^.

The use of gloves causes a significant reduction in nicotine absorption, reflected in the low rates of excretion of nicotine and cotinine^25^ and the consequent reduction in the disease’s symptoms. In a study^24^ where the farmers did not wear boots or rubber gloves during work and worked in wet conditions, there were subjective symptoms such as nervousness, dizziness, pallor, rash, numbness and muscle weakness.

In another research project carried out in the central and southern regions of Rio Grande do Sul, the efficacy of a standard uniform with impermeable fabric (long-sleeved shirt, pants and nitrile gloves) was evaluated during the harvesting of Virginia tobacco leaves. The results indicate that the uniform provided around 98% protection^25^. Regarding the use of socks as a risk factor for GTS, there is no consensus in the literature, as it is referred to both as a risk and as a protection factor^13^; the risk factor is existing because the high temperatures in the tobacco harvesting period, combined with wet tobacco handling and the use of boots and socks, increase sweating and perspiration, favoring transdermal nicotine absorption^6,9^, as well as the hypothesis of absorption through the feet^9^. In contrast, the protective effect would be the use of socks, associated with other protective equipment, which would reduce the exposure to nicotine and, consequently, the chances of developing GTS^13^.

The measurement of cotinine based on biological markers allows more accurate estimates. Both nicotine and cotinine can be screened in urine, blood and saliva, organic materials that are easier to use routinely^14^. Urinary cotinine, in addition to being easier to collect, has cotinine concentrations that are four to six times higher than other forms^26^.

In this study, the results of the cases’ urinary cotinine examinations (median of 114.9 ng/mL) suggest the presence of GTS. The values shown by some control workers that were above the reference value indicated by the laboratory may be related, according to evidence published in a study^24^, to the dermal absorption of the nicotine contained in tobacco leaves. The median urinary cotinine among the case workers was higher than that of control workers (98.5 ng/mL), as well as the one shown by nonsmokers. The results presented here are similar to data from the literature^10,24^.

In a study^5^ developed in South Korea, with five urine collections at different time intervals, higher cotinine concentration was identified in the morning samples (mean of 500.71 ng/mL), and lower concentration was identified in the idle period after dinner (mean of 135.40 ng/mL). In the study in which GTS was described for the first time in Brazil, with rope tobacco workers, the median of the cotinine levels found in the urine of the smoking case workers was 811 ng/mL, while for the control, it was 1,293 ng/mL; among the non-smoking cases, it was 288 ng/mL and for the control, it was 156 ng/mL^10^. In a study carried out in the region of Vale do Rio Pardo, Rio Grande do Sul, the mean cotinine levels in the urine of Virginia tobacco workers was 432 ng/mL for the cases and 353 ng/mL for the controls^8^. In the northern region of Rio Grande do Sul, cotinine levels ranged from 20.5 to 515.0 ng/mL among non-smoking workers^27^.

The difference in urinary cotinine levels may be associated with differences in the methodologies used by the studies, as well as, according to other studies^5,27^, to the time of exposure to tobacco, ethnicity-related differences, urine collection time and smoking, as well as the action of enzyme CYP 2A6 (cytochrome P-450, Family 2, Subfamily A, Polypeptide 6), which is responsible for the degradation of the nicotine in the liver^27^ and distributed in blood, saliva and urine^26^.

The high urinary cotinine dosage among the control workers was also described in a study carried out in Brazil, which suggested the dermal absorption of the nicotine contained in tobacco^10^. The consequences of contact with the nicotine in tobacco in the medium and long term have not yet been explored^1^.

Given this context, it is possible to perceive a socio-environmental relationship in which the work process of workers in tobacco cultivation and their working conditions influence these workers’ health and sickness, as well as the ecosystem’s functions as a whole, with consequences also for the environment. The tobacco plant itself is a source of risk compared to other crops, and the various tasks involving tobacco production expose workers to the risk of developing GTS^28^.

As a limitation of the study, it should be noted that non-participant observation was not performed, which would allow monitoring the workers’ exposure to risk factors and sickness during the work process. However, it is expected that the evidence found enhances the interest in new and necessary studies in the area, with analyses that offer a clearer categorization of the determinants related to the specificities of the work process and environment and their relation to the disease, including prospective studies to assess biological and external factors that may influence it.

## Conclusion

The results of this research may suggest the presence of GTS in Burley tobacco workers, and the use of the biomarker technology was appropriate and extremely relevant for the evaluation and elucidation of the suspected cases. Thus, it is important to emphasize to municipal managers the need of making this technology available and including it in Primary Care, since its cost-benefit allows offering the confirmatory examination to workers and health professionals.

The action of health professionals and professionals in other fields (agricultural) is necessary for the development of preventive actions on the disease and to make workers aware of the importance of the use of gloves and impermeable protective clothing, as well as of the proper management of the plant in a suitable period, so as to avoid factors that contribute to the development of GTS, such as working in wet conditions (wet tobacco and wet clothes).
